# Ready for Her Close-Up: An Interview with Elaine Strass

**DOI:** 10.1371/journal.pgen.1000002

**Published:** 2008-02-29

**Authors:** Jane Gitschier

**Affiliations:** Departments of Medicine and Pediatrics, Institute for Human Genetics, University of California San Francisco, San Francisco, California, United States of America

**Image 1 pgen-1000002-g001:**
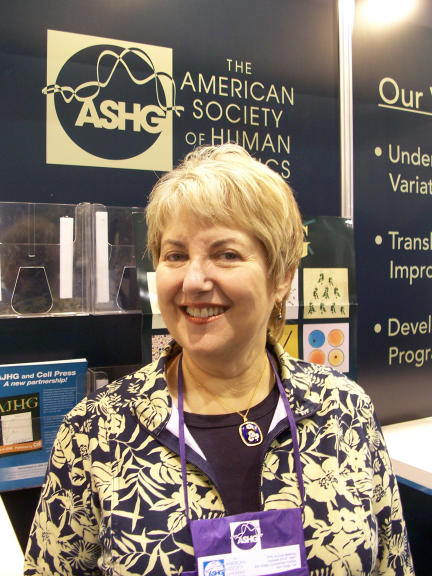
Elaine Strass.

When I invited Elaine Strass for an interview, I had no idea she was planning to retire in the coming year. Elaine has been the hidden force behind both the Genetics Society of America (GSA) and the American Society of Human Genetics (ASHG) for almost 20 years, by serving as Executive Director for both organizations. Fresh, articulate, and cheerful, Elaine (see Image 1) has a lightning wit, great people skills, and zest for her work. She is our societies' strongest champion, yet many of you may be unfamiliar with her.[Fig pgen-1000002-g001]

To catch up with Elaine, I flew to the ASHG meeting in San Diego in late October with a bit of trepidation and a lot of sadness for the residents there, as fires were devastating the outlying areas. From the window seat on my evening flight, I easily spotted at least half a dozen blazes in the distance. It was a spooky sight, reminding me of the strong natural forces that shape our planet, but I couldn't help likening the vista to red fluorescent probes beaded along interphase chromosomes against the black landscape of a FISH experiment.

Unannounced, I located Elaine the following morning at the ASHG headquarters in the convention center. We popped a few batteries in the recorder, turned on the machine, and we were off—literally! At a quick pace and with effervescent description of the success of the meeting so far, she swept me along to show me the new booth that ASHG had designed to advertise the next International Congress of Human Genetics. She was ready for her close-up.

**Jane Gitschier:** How long have you been the executive director?

**Elaine Strass:** I became the executive director of ASHG and the Genetics Society of America in 1992, when Gerry Gurvich, who had started the Washington office in 1983 for the two societies, decided to retire. It was the first time either society had had an office, and they decided to do it together for economies of scale. They shared staff and that is still true to this day.

**JG:** How did you land this job?

**ES:** Well, I started off as a concert pianist, and I wasn't very good and I needed to get a day job. The only thing I really knew how to do was type. So I went back to school and learned a little bit about computers and word processing, which was *the* thing then. I was taught on a Wang system.

I got various jobs doing word processing. I was staying home with my kids, so I wasn't working full time, but I was having a wonderful time, and I was also doing a lot of concertizing in the community.

**JG:** In Washington?

**ES:** Yes, but I was never very famous. You have to put this in perspective, Jane. I'm a very good pianist, but I'm not a GREAT pianist.

**JG:** What kind of concertizing did you do?

**ES:** I was the official accompanist for the State of Maryland for competitions they had, like violin concertos, cello concertos.

**JG:** Had you been a conservatory student?

**ES:** I was a graduate of the University of Illinois. Bachelor of Music in Performance Piano. I *loved* playing the piano. I did accompanying stuff, pianist for shows. It was a lot of fun for me.

Anyway, to get on with my brilliant career. … When my third child was in second grade, I went to work part-time for a law firm in Rockville.

Then, in 1981, I heard about a job that I really wanted. It was for the Society for Neuroscience, and it had to do with computers, sessioning for abstracts for their annual meeting. They were panicked because they had 5,800 abstracts, and they had never seen so many abstracts in their lives! This was the first year that they were going to be using a computer. They hired me.

That is also where I met Gerry Gervich. We worked together for about three years. And later Gerry met some geneticists—including Art Chovnick, who was the most instrumental in putting together that first [genetics] office. They got the idea that ASHG and GSA would chip in and have employees and then everything would be official—the registration, documentation, computers for membership. Gerry had this all mapped out in her mind. She wanted to hire me then, but she didn't have the budget.

Then Gerry called me one day and said, “I've got the budget, I'm going to hire you.” So in 1988 I started working for ASHG, but not yet for GSA—raising funds, doing committee work, and supplementing what she had already set up. I loved my job; I was so happy. I loved working with the geneticists and I became fascinated with genetics, even more so than I had been fascinated with brain science.

After several years, Gerry decided to retire. She asked me if I would take the job. I didn't think I had the right stuff. It's a really tough job. Gerry had a lot of faith in my ability, and both boards [of GSA and ASHG] agreed that they wanted me. The first meeting I did was the San Francisco meeting of ASHG.

**JG:** Do you go to the meetings?

**ES:** I go to all the meetings. I go to the yeast meetings. I go to the *C. elegans* meetings, which we've started to do. They meet every other year. They usually meet at UCLA. As long as we can do it this way, we will, but some of these meetings are threatening to grow even more, so we may not be able to do the campus meeting.

The *Drosophila* community has a meeting once a year and they have grown to such a size—there are 1500 people now—that they meet partly in a convention center, partly in a hotel.

But some of the other meetings are less sizable, such as the yeast meeting, which has between 800 and 900 people every other year, and what they prefer to do is to meet on a college campus in the summer and to stay in the dorms. The cost is so reasonable for the students who go. It's a good deal.

GSA handles all these different kinds of meetings. There's also fungal genetics, about 800 people and they meet at Asilomar every year, but they may have to change their venue because of their growth.

**JG:** What about the zebrafish community?

**ES:** Zebrafish is going to contract with GSA and have their meeting organized by GSA in two more years.

The campus meetings are great, but the problem is that when you start to have too many people coming, you really need to be in a tax-free situation where you can receive money and not pay income tax on the receipts from the registration fees. So you need a tax-exempt carrier for the money. And that's why GSA became so important in that community, with all those little organismic meetings, which are extremely critical to the development of those fields.

**JG:** I assume GSA has their own president and board of directors?

**ES:** When Gerry Gurvich put them [GSA and ASHG] all together in 1973, she modeled them very similarly. The idea was to keep them totally separate, because they do not have very many overlapping members, and they have separate goals.

The governance structures are pretty much the same. The main difference is that GSA, in their election process, has two people running for president, but ASHG has only one. Of course that is always being discussed by the ASHG board: Is that the best way to represent the society? There are ways to look at it from both sides.

The nominating committee is wonderful—ASHG and GSA both have these committees and they take their job very seriously. We always have very good leaders who are dedicated to the societies and their missions. It is always a wonderful thing to see this all unfold with their elections.

The argument for having one candidate only is that [with a two-person race] you can work your way through a community generating non-winners, and sometimes there are hard feelings. We really don't want to do that.

People work themselves up through the ranks of ASHG, we don't ever nominate a president who isn't familiar with the work of ASHG, who hasn't been to the meetings and participated in the work of the society. So we are very familiar with a candidate and truly recommend someone who is just great. And we keep lists from year to year, so sometimes, when a name gets on, it might take five years until they work their way to the top, so they really have been elected in a sense. It's a very thoughtful process and I recommend keeping that. I think if people knew the process, they would agree that it is a good one.

**JG:** What is the membership of GSA?

**ES:** Right now 5,100.

**JG:** And ASHG?

**ES:** 7,200.

**JG:** Tell me exactly what you do?

**ES:** My job is very exciting. I run the office in Bethesda. We have 14 employees. We are a very well-oiled machine. Our IT department is our biggest department. Everything is done over the Web—dues, memberships, meetings.

One of the most important parts of running a non-profit organization is to document all financial transactions. And then there is the paying of bills for the meetings. ASHG meeting here in San Diego, for example costs $2M. We don't really make much money from the meetings.

I make sure things happen. I listen to what the board wants. I give them my ideas. Sometimes my ideas are very well received. Sometimes I have to re-introduce an idea several times.

People come to me and I serve as a funnel, and I see that as a very important part of my job. Also, when I read something in a magazine or newspaper, or if I get an idea from the Internet, as the Executive Director, I have the authority to institute a lot of ideas.

Right now it's a wonderful time for us to be doing this because of the Internet. It has changed the way societies do business and has made everything much cheaper to run and extremely efficient. We're delighted.

We never take Fedex submissions of abstracts—we used to have 2,000 Fedex envelopes all arriving on the same day at the office! The fascinating part was the fear we faced—oh, we'll never be able to do this electronically! What if the disk breaks, or something? It's in the ether, it's not concrete. But we made that transition very, very quickly.

**JG:** I assume you like your job.

**ES:** I love it!

**JG:** Why?

**ES:** First of all, working for geneticists is for me a big thrill. Don't forget, when I was a housewife sitting in my garage watching people drive up and down my street, I didn't see many Nobel Prize winners!

But I do now!

And one of the biggest thrills at GSA this year has been the realization that GSA has been making awards, and the recipients then go on to win the Nobel Prize. The GSA Medal went to Bob Horvitz and then he won the Nobel, and then to Andy Fire, and he won the Nobel. John Sulston was another awardee, and then he won the Nobel. This year we awarded Oliver Smithies, and he just won the Nobel! We beat the Nobel to it! It made us feel terrific.

The GSA has three awards traditionally. There was the Thomas Hunt Morgan Award, which is a medal and it's been given since the early 60s, and there is a picture of a fly on the medal and on the other side is Thomas Hunt Morgan's portrait—4.5 inches in diameter, made out of pewter. And this is the big one. You get this award when you have shown lifetime contributions to the field of genetic research.

The GSA Medal was developed a few years later. Why should we have to wait for a whole lifetime for contributions to occur before we can honor someone? The idea for the GSA Medal was a breakthrough within the last 15 years. And that was the one that Horvitz won, for apoptosis, programmed cell death. He had other things, too. It was not difficult to give Bob that award!

And Oliver Smithies, of course, for his great contributions—he got the Thomas Hunt Morgan Medal for lifetime contributions.

**JG:** So the GSA Medal is like the Curt Stern Award that ASHG gives.

**ES:** Yes, it's the parallel to that.

GSA wanted to honor people, starting about six years ago, for service to the community. It's called the George W. Beadle award. We have people who have made enormous contributions, who have made the lives of the scientists so much easier and even possible. Like, if you ran a stock center for 20 years, you might not get a scientific award, but, my goodness, your donation to the community is so enormous! So that's why the Beadle Award was developed and every year we come up with some really great winners.

**JG:** With GSA having all these separate meetings, how do they coalesce to make decisions about things?

**ES:** It's a good question. Let's take a step back and look at the bigger picture. We have different models for societies.

We have Society for Neuroscience with 48,000 members. When they have a meeting and 34,000 people come, the city knows that they are there because they have taken every single hotel room. They have so many poster presentations that they have a complete poster session in the morning, then they take it down and another complete one goes up in the afternoon, and that goes on for five days. The scale is different from both ASHG and GSA.

There is a drawback there because when you go to meeting of that size, it's hard to run into people. They have specialties, too, but they elected to keep everything together. I remember when I worked there, there was always the threat that the behavior neuroscientists would split off! “We don't like the way the board is treating us and we're going to split off!” But they never split off.

Still you know where you fit in, your little corner in the very large meeting.

ASHG is kind of in the middle. We get together once a year, we know we can bump into people we want to bump into. We have little cubbyholes to leave messages.

However, we also have American Society for Gene Therapy, NSGC (National Society of Genetic Counselors), HUGO (Human Genome Organization), the American College of Medical Genetics—all these other groups who are very close to ASHG. But the ASHG meeting is *the* research meeting, and we have chosen not to do the neuroscience route, which is to include everyone at one meeting.

The way I interpret it—genetics is nature's way of making diversity, and that's what we've got in the genetics community.

All these organism guys, they really don't want to meet with each other. The yeast guys don't want to meet with the worm guys and and they don't want to be with the fungal guys. They call *Clamydamonas* guys the “pond scum” guys. The Drosophilists have their own wonderful community. They have their own board of directors, even though they are not incorporated. It's very loose, they don't have to file reports.

So the question is—could there be a really large model organism meeting? It would be about 9,000 people, and I've given this a lot of thought. Possibly serial overlapping meetings. But nobody is hot to do that. They like their small meetings, which are very predictable, good ways for students to present their first poster or talk, and to teach students how it is done—how to meet the right people and publish.

**JG:** So, with all these independent meetings, how does GSA come together?

**ES:** There is the journal *Genetics*. GSA was started in 1931 and the journal in 1916! And all of those old issues are on the Journal Web site through Highwire Press. So the Journal acts like a coalescing factor. And the GSA board has been discussing this for years, and that why they came up with the Model Organism to Human Biology (MOHB) meeting, which meets in San Diego every other January.

This was actually a decision made while Mark Johnston, a yeast researcher, was president of the GSA, and he happens to be a very visionary person. He, along with many others on the board, the Drosophilists, thought it was time to bring things together. Part of it had to do with the fact that everything is getting sequenced, and this made a huge difference in the way we look at genetics research and the future as geneticists and genomicists. There's a lot more that we have in common because of these important features. And the Journals acknowledge this, but having a meeting where you have the human and the Drospholist and all talking about the same gene—in yeast and worm.

At the last MOHB meeting, I was floored.

**JG:** So you went to the sessions?

**ES:** I always go to the sessions. I don't know how I can understand them, because I'm not trained as a geneticist. To me, genetics is the mechanics of the tinkertoys of life. It's a huge puzzle and I *love* the idea that it could be decoded, that it could be sequenced. The idea that somebody would invent PCR. I understand enough to see the wonder of all this.

**JG:** You're tearing up!

**ES:** I'm just an old sentimental fool.

To see the changes in the field! I'm almost an outsider. I don't understand enough to really appreciate everything that's going on, but when somebody gets the Nobel Prize, I understand.

**JG:** Why are you retiring?

**ES:** Well, I'm more aged than I look! I've got old arteries. I have to take care of myself. This is a big job. A $9M budget—two budgets! And a lot of travel, if you go to all the meetings. I go to everything—I wouldn't miss anything—it's too exciting! And that's a problem I have; if I could step back and not be as involved, it would be better for me. I can't seem to do that! So, I announced two years ago that I was going to retire.

There are other things I want to do. Go back to music and work in the community. I'm starting a business called “Dial-a-Daughter.” I will take old people who can't drive any more who want to do things with me, like go to the opera or out to lunch. I will devote my time to helping these people *live*.

This will be my new life, and I of course will continue to be a member of GSA and ASHG.

There are so many people who have helped me—these people are wonderful. I adore geneticists! Warm people who just helped me understand anything I didn't understand and helped me get through the things I wanted to get through. This has been a real “love in,” for all these years.

For me, it's the end of an era, and when I look back on my life, this has been the best part of it. There is no question that what I have here is rewarding and exciting—it's a dream job, and it's all because of these two societies.

